# The Adaptation and Feasibility of Narrative Enhancement and Cognitive Therapy (NECT) for Late-Onset Psychosis

**DOI:** 10.1007/s10597-019-00495-5

**Published:** 2019-10-29

**Authors:** Elaine Y. N. Ching, Lucy Smyth, Tanisha De Souza, Georgina Charlesworth

**Affiliations:** 1 Research Department of Clinical, Educational and Health Psychology, University College London, Room 436, 4th Floor, 1-19 Torrington Place, Gower Street, London, WC1E 7HB UK; 2 Research and Development, North East London NHS Foundation Trust, Goodmayes Hospital, 157 Barley Lane Ilford, London, IG3 8XJ UK; 3 Barnet Psychology Hub, Barnet, Enfield and Haringey Mental Health NHS Trust, Springwell Centre, Wellhouse Lane, Barnet, EN5 3DJ United Kingdom

**Keywords:** Late-onset psychosis, Psychological intervention, Narrative therapy

## Abstract

The aim of this study is to adapt and feasibility test the narrative component of Narrative Enhancement and Cognitive Therapy (NECT) for late-onset psychosis. This study followed the development and feasibility phases of the Medical Research Council framework. The original NECT intervention was adapted based on consultations with service users, experts, and clinicians. The evaluation of the feasibility test of the adapted intervention was guided by Orsmond and Cohn (Occup Particip Health 35(3):169–177, 2015)’s model for feasibility studies. The final adaptations consist of language, readability, and delivery. The adapted intervention was tested for feasibility and acceptability with one group of five patients recruited from a National Health Service (NHS) Trust in UK Results were mixed in participant outcomes and a likelihood of acceptability of the intervention. This indicates the need for a larger scale feasibility test to explore the identified benefits and challenges of implementing NECT in NHS or community settings for late-onset psychosis.

## Introduction

### Psychosis and Recovery

Late-onset psychosis (LOP) is a condition characterised by a ‘loss of contact with reality’, with an onset after the age of 40 (Howard et al. 2000). The current recommended treatment for all ages is a combination of oral antipsychotic medication and psychological interventions, namely Cognitive-Behavioural Therapy for psychosis (CBTp) and Family Intervention for psychosis (FIp) (CG178: NICE 2014). However, results from investigations of these treatments have been inconclusive (Jobe and Harrow 2010). Researchers have thus shifted away from symptomology as a treatment outcome measure, and towards a recovery model which emphasises social and psychological recovery from mental illness (Bonney and Stickley 2008; Lysaker and Buck 2008; Bellack 2006). Elements of social and psychological recovery include hope, identity, sense of self, and empowerment.

### Narrative Approaches to Challenge Stigma in Recovery

A major barrier in the recovery process is stigma (Wilken and Hollander 2005). Stigma attaches a negative and distorted identity to the affected individual (Goffman 1961) causing significant impacts on their life. Narrative approaches understood this as a process of stigma and ‘othering’. Society constructs a perception of the ‘norm’. People who deviate from the norm are classified as ‘others’ and treated by society based on this tainted narrative rather than their original narrative. Some may internalise these negative narratives about themselves, termed ‘self-stigma’. Over time, individuals may view their past and future in terms of failure and inability (Lysaker et al. 2007), leading to loss of motivation and feelings of disempowerment. As they begin to withdraw from society and adopt the sick role, their persistent symptoms and dependency on carers and the mental health service are further reinforced, hindering their recovery. Lysaker et al. (2007) found that people with a diagnosis of schizophrenia who were classified with low functioning demonstrated high levels of internalised stigma and low levels of insight.

The narrative approach proposes the use of collective storytelling as a way to resist silencing. When marginalised groups meet and share stories, they can begin to claim control and authority. Sharing the unheard and hidden stories may expose cracks in the dominant narrative that society has given them. This allows a new, alternative and coherent narrative to emerge (Andrews 2004; Fivush 2010). The process of telling stories, being heard and validated by listeners is key to reducing and resisting stigma and self-stigma (Prasko et al. 2010; White and Epston 1990). It also allows individuals to find meaning from their experiences, communicate and earn validation from others which helps them move forward in recovery.

### Narratives in Late-Onset Psychosis

Narratives and stories have been used in many cultures. Stories are a way to pass knowledge collected through life experiences from older to younger generations (Eder 2007). Reviewing past experiences is a natural process for humans and relies on one’s autobiographical memories and the meanings and emotions which are attached to the memory. The therapeutic benefits of reminiscing are well established. Butler (1963) reported that older psychiatric clients reminiscing achieved ‘resolution of old conflicts, personality reorganization, and restoration of meaning in the individual’s life’. Bohlmeijer et al. (2008, 2009) suggested that the reminiscence process helps individuals take agency for their preferred ways of living within their life circumstances. A meta-analytic review of 128 studies of reminiscence interventions, including autobiographical writing and life story work, identified moderate impacts on psychosocial outcomes such as ego-integrity, mood state and purpose in life (Pinquart and Forstmeier 2011). However, there are no existing evaluations of narrative interventions with people with late onset psychosis. A qualitative study with individuals with LOP (Quin et al. 2009) indicates the potential value of a narrative approach. Although participants experienced negative feelings when reviewing their life (e.g. feeling alone and different), they also shared meaningful, narrative accounts and explanations for their psychotic experiences.

### Narrative Enhancement and Cognitive Therapy (NECT)

NECT is a 20-week group intervention aimed to help individuals with severe mental illness (SMI) recover through recognising self-stigma, exploring unhelpful beliefs, and creating an alternative self-narrative (Yanos et al. 2011). Participants engage in a series of story-telling exercises where they are encouraged to share their experiences and receive feedback from facilitators and participants. The intervention also uses psychoeducation to increase participants’ understanding of mental health to reduce self-stigma, and cognitive techniques to equip individuals with the metacognitive capacity to narrate. The developers proposed that the key mechanisms of change lies in the interpersonal context, the process of sharing and narrating with others, and emotionally-present facilitators. They suggested that story-telling helps individuals shift the meanings they attach to their experiences and self through their relationship with others in the group (Roe et al. 2010). They move from being undermined by societal stigma to being heard and validated by others (Geekie and Read 2009).

There is a small yet growing evidence base on the effectiveness of NECT. The manual was piloted in the United States (Roe et al. 2010; Yanos et al. 2012), Israel (Roe et al. 2014), and Sweden (Hansson and Yanos 2016; Hansson et al. 2017). It was delivered to individuals with schizophrenia, schizoaffective, bipolar disorders, and major depression. Results consistently showed reductions in self-stigma and improvements in insight, self-esteem, quality of life and hope. This was reflected in qualitative feedback of experiential learning, positive change in the experience of self, acquiring cognitive skills, enhanced hope, and coping and emotional change. Also, studies have found benefits of individual modules (Bossema et al. 2011; O’Driscoll et al. 2016; Lysaker et al. 2001, 2005, 2007).

### Adaptation of NECT for LOP in the United Kingdom

Given the close fit between reminiscence therapy and narrative approaches, the narrative component of NECT may have particular relevance to people with LOP following adaptation for the UK context. Singling out the narrative module allows examination of its effectiveness, given the existing evidence for the CT component in NECT, evidenced from CBTp.

### Aim and Research Questions

This study aimed to adapt and feasibility test the narrative component of NECT (Yanos et al. 2011) for adults with LOP (age 40 and over), following the commonly used Medical Research Council (MRC) framework for complex interventions (Craig et al. 2008). Frameworks were used to enhance methodological rigour which is essential during the early stages of intervention development (Hoddinott 2015).

The research questions are:

1. What adaptations are required to apply the narrative module of NECT for people with LOP?
2. Is the adapted module of NECT acceptable by people with LOP, their family/friends and clinicians?
3. Are outcome measures of NECT acceptable by people with LOP, their family/friends and clinicians?
4. Is it feasible to deliver the adapted module of NECT within an outpatient NHS setting?
5. Can the adapted module of NECT yield change in participants’ clinical, social and psychological recovery?

## Methods

### Research Design

**Fig. 1:**
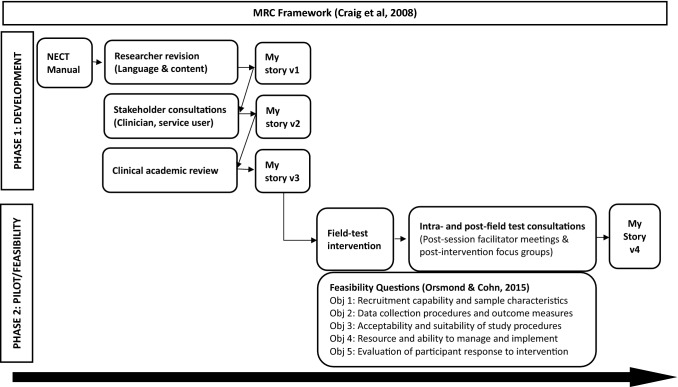
Adaptation consultations and Feasibility Guidance within the MRC framework

This study adopted a mixed methods approach, following the two stages of the MRC framework. The first phase is the development of the intervention manual, guided by consultations with key stakeholders. The second phase is testing the adapted intervention for feasibility and acceptability. Feasibility refers to whether the intervention can be conducted, and acceptability refers to whether the intervention is accepted by the target population. This process was evaluated using Orsmond and Cohn (2015)’s guideline for feasibility studies (Fig. 1).

### Phase One: Development and Adaptation

**Fig. 2:**
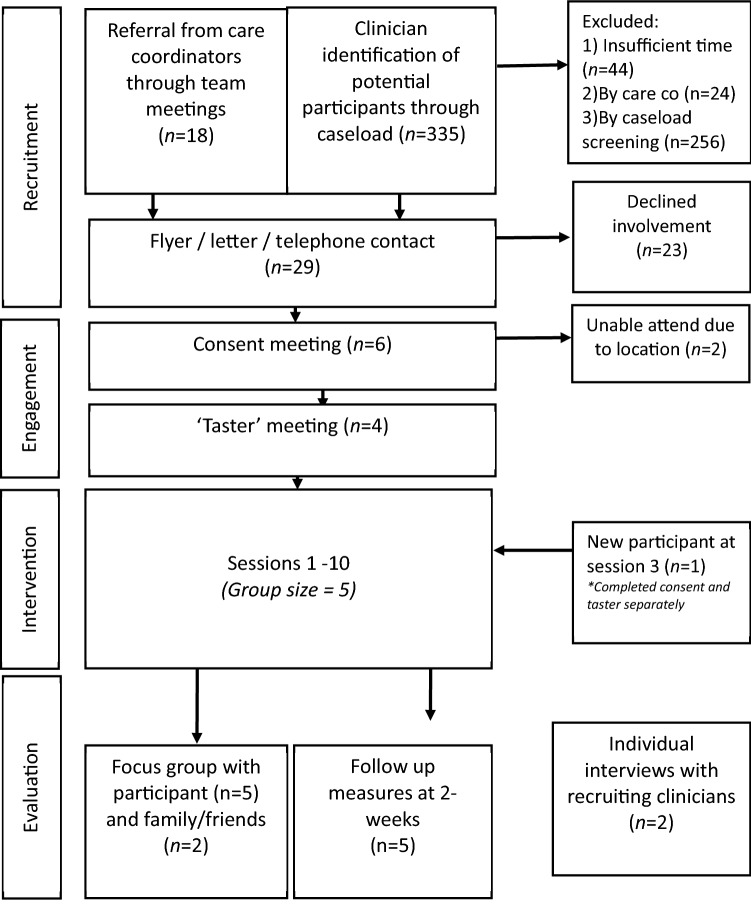
Process from recruitment to follow-up

The current study based adaptations on suggestions made by the key stakeholders of the intervention via consultation meetings. Key stakeholders include researchers, clients, family members/carers/close friends, and NHS clinicians. First adaptations were made to the written language to adapt to the UK, and literacy levels of the target population using the Flesch-Kincaid Grade rating scale and Flesch Reading Ease rating scale. Further adaptations were based on consultations on four aspects of the intervention: manual structure, manual content, administration, and treatment setting and location (Fig. 2).

### Phase Two: Feasibility

The adapted manual (My Story version 3) from phase one was delivered in ten 90-min weekly group sessions by NHS clinicians as an optional standard care service for individuals with LOP. The group was led by one consultant clinical psychologist (GC), co-facilitated by one clinical psychology trainee (EC), and further support from an assistant psychologist (TD). The process from recruitment to follow-up was evaluated using the feasibility guidance by Orsmond and Cohn (2015). This guidance consists of a series of questions to address the five key objectives of a feasibility study.

#### Objective One: Recruitment Capability and Sample Characteristics

Participants were recruited from NHS secondary care services based on two criteria: age 40 years and older (the cut-off age for LOP (Howard et al. 2000)); and a formal diagnosis of schizophrenia, schizophreniform, schizoaffective, delusional disorder, brief psychosis or psychotic disorder not otherwise specified.

Two recruitment approaches were used, one involving a ‘link clinician’ who discussed the research at team clinical meetings, and the other involving a clinician screening the team caseload. Upon referral, clients would be contacted via telephone or post and briefed on the details of the study by the researcher. Participants who expressed interest were then invited for an initial meeting to discuss questions about the study information in detail, and provide written consent. They were then invited to attend a ‘taster session’ with the researcher to complete a sample exercise from the manual, a safety plan and to answer any questions about the group. Successful recruitment rates were calculated and analysed.

#### Objective Two: Data Collection and Outcome Measures

The following questionnaires were administered at each session, accompanied with the estimated completion time.:

1. Questionnaire on the Process of Recovery (QPR; Neil et al. 2009)
2. Brief version of the Internalised Stigma of Mental Illness Scale (ISMI-9; Hammer and Toland 2016)—1–2 min (Boyd et al. 2014).
3. Birchwood Insight Scale (Birchwood et al. 1994)—5 min completion time (Waters and Stephane 2014)
4. Brief Symptom Inventory (BSI; Derogatis and Melisaratos 1983)—8–10 min (Brief Symptom Inventory 1993)

Participants’ feedback on outcome measures were recorded via researcher field notes after each session, and/or post-intervention focus groups. Time taken to complete the questionnaire were also recorded.

#### Objective Three: Acceptability of Intervention

To examine participants’ acceptability of “My story”, attendance rates and session ratings were collected weekly. The Session Rating Scale (IAPT SRS; Miller et al. 2000) was used as a measure of satisfaction with each session. Field notes on engagement and safety were also taken during each intervention session to gather data on group dynamics, themes of discussions, engagement and other practical issues (attendance, risk). Acceptability were based on the criterion: SRS ratings > 20; Attendance rate > 50%; participation and engagement during sessions (field notes).

#### Objective Four: Feasibility of the Intervention

Feasibility of the intervention was evaluated using the following criteria: recruitment of at least one group of five to six participants; sessions could be delivered without disruption; minimal amendments to manual i.e. retained session topics.

#### Objective Five: Evaluation of Participant Response to Intervention

To examine whether the intervention showed a likelihood of success for the targeted population, quantitative outcome measures and qualitative field notes of sessions (See procedure of objective three) were analysed and triangulated with post-intervention qualitative interviews with participants, family/friends and referring clinicians (See clinician interview of objective one). The study adopted the mixed methods multiple single-case design/analysis to allow for optimal investigation of within-subject variability across time and for a small sample size. Outcome scores were inputted and analysed using Statistical Analysis Software Package (SPSS v21). Outcome scores were prorated to account for missing values. A three-step method was used to analyse multiple single-case studies (Borckardt et al. 2008): (1) Graphical display of change over time (Parsonson and Baer 1992); (2) use of clinically significant change (CSC) and reliable change (RC) and; (3) qualitative data from participant follow-up focus groups or individual interviews.

#### Ethics Approval

This study was approved by the NHS Health Research Authority—East Midlands—Leicester Central Research Ethics Committee (IRAS Project ID: 194877, REC reference: 16/EM/0275).

## Results

### Phase One: Development and Adaptation

The initial adaptation involved the extraction of materials, including introduction and ending sessions, and the narrative module. Terminology (US to UK) and readability were also adjusted for the target audience. This adapted ‘My Story’ v1 was then circulated to four service users and nine clinicians for review. Adaptations based on these suggestions were made, including replacement of terms which were felt to have a negative connotation/stigmatising; providing alternative communication of stories; duration of sessions. Version 2 was further reviewed by three clinical academics, who suggested final amendments to the readability of the materials. Adaptations were made to produce ‘My Story’ version 3.

### Phase Two: Feasibility

#### Objective 1: Recruitment Capability and Sample Characteristics

Firstly, we examined recruitment success rates. The final recruitment rate based on the number of referrals and caseloads screened is 2%, of which 7 of 18 clients approached agreed to participate. Of seven sites, only one site recruited the sufficient number of participants to form a group.

Secondly, we examined the challenges and barriers during the recruitment process. Sites adopted one of two recruitment approaches. Firstly, an identified ‘link clinician’ at the site to promote the project to their team, accompanied by the distribution of recruitment flyers via email. Link clinicians identified that the key barriers to recruitment were clients’ general engagement with mental health services; openness to using psychological therapies; stigma as a barrier to help-seeking; physical mobility; and capacity of the clinical team. The second recruitment approach involved researcher screening caseloads with each care-coordinator. One-site succeeded in screening all caseloads. 27% of the team’s full caseload were screened by researchers and deemed to meet the eligibility criterion of LOP in the absence of any progressive cognitive impairment. Care coordinators further screened and identified eleven clients. However, all clients declined. Care coordinators and clients suggested that key barriers to recruitment were location and poor physical mobility; stigma about psychosis; insight about condition; isolation; risk; and length of therapy for the proposed group.

Thirdly, we examined the relevance of the manual for recruited participants. Demographics match that of the targeted audience, which showed a balance of male and female, with an age range of 41–60 years, and a mixture of ethnicity. Only one participant was employed. All participants had received support from psychological services or individual CBT (cognitive behavioural therapy) before attending the group.

#### Objective 2: Data Collection and Outcome Measures

All participants except one completed the full set of measures at all collection points. Based on session field notes, participants took 15–20 min to complete measures, which is consistent with the estimated completion time. Four key themes were identified from participants’ feedback. This includes the impact on emotions e.g. feeling upset by questions; high frequency of collection and time consuming; understanding of questions; and relevance of questions to their needs. It appears that apart from meeting the threshold for the feasibility of the intervention such as the completion time, other aspects for the acceptability of the interventions such as willingness to complete the questionnaire were not met.

#### Objective 3: Acceptability of Intervention

All participants completed the 10-week intervention and the mean attendance rate was 8.4 sessions (86%). Full attendance was achieved for sessions 7, 8, 9 and 10, and lowest at session 5 and 6. Reasons for non-attendance were: clashing appointments, lateness, childcare, and work. No adverse events were noted.

Participants were observed to adhere to and engage with group sessions. Some participants initially expressed feeling uneasy and feared their story was not ‘good enough’ but were observed to share experiences and encouraged each other after session seven. Two clients did not agree with their diagnosis but were open to engage in group discussions. All participants felt comfortable with writing. Finally, two clients required telephone support after feeling emotional in session three. It was noted that both clients were experiencing ongoing difficulties outside the group setting.

Participants rated highest satisfaction scores for session five and ten and lowest for session three, with mean scores of 35. Scores were consistent with qualitative feedback which was collected 2 weeks after the final session. Feedback was positive towards the routine of sessions; group size; group setting; use of materials e.g. flipcharts; and in particular, sharing and hearing about experiences. Suggestions were made towards increasing the number of sessions as group identity took time to establish and to the sensitivity of topics given each member are at different points in their recovery.

It appears that the intervention is highly acceptable, with SRS ratings over 20, attendance rate over 50% and a general positive field observation of participation and engagement.

#### Objective 4: Feasibility of the Intervention

Facilitators shared positive feedback regarding sufficient physical space, administration support, materials, staff expertise and skill; within budget; timely data-input; and appropriate operation policy in managing distress. Facilitators also commented on the observed distress in participants during session three during the exercise where participants were asked to generate words to describe their mental health experiences. Facilitators expressed that despite distress which was addressed outside the session, the exercise itself gave the first opportunity since the start of the group for members to learn that they have a shared experience (Table 1). Facilitators met between sessions to make ad-hoc changes to the manual to increase ‘fit’ with the client group. Facilitators commented on the during recruitment stages.

**Table 1:** Description of delivered sessions

Session	Topic and session summary	Attendance ratio (attenders: non-attenders)	Session satisfaction (SRS total: mean, range)
1	*Introduction to group* Orientation to group aims and rules. Discussions on mental health, employment and stigma	4:0	31 (23-38)
2	*Identity and labels* Continued discussions from session 1. Exercise on an identity that was important for participants. Discussions on employment, stigma and negative emotions	4:0	34 (31-38)
3	*Exercise 1: Stories of self:* *‘Words to describe your mental health experience’* Discussion on an article brought by a participant about stigma and mental health. Exercise on sharing words that described their mental health experience. Discussions on response to their experience (shock); emotions (sadness, anger); and sense of support (being alone, stigma)	4:1^a^	30 (22-38)
4	*Exercise 2: Stories of self: ‘Importance of sharing’* ‘Circle of friends’ exercise to identify individuals within their network they feel comfortable to sharing their experiences with. Discussions around self-stigma as a barrier to sharing, individual’s determination to seek help and the importance of talking as a way to make sense of experiences	4:1^b^	36 (35-37)
5	*Introduction to stories/narratives* Discussion on story genres and preferences, ownership of narratives on their mental health experiences and the creation of alternative narratives	3:2	38 (36-40)
6	*Exercise 3: Stories of coping: ‘getting here’* One participant shared the quote ‘we cannot repair the past but we can build the future’. Participants identified themes from their stories on the topic: hope, determination, willingness, openness, strength, courage, perseverance, control and choice	3:2^b^	36 (33-40)
7	*Exercise 4: Stories of strengths and weaknesses: ‘daring to share’* One participant shared their story about facing fears of others’ view. Themes identified were courage and determination	4:1	37 (34-40)
8	*Exercise 5: Stories of strengths and weaknesses: on ‘daring to share’ (cont’d)* Discussion on the importance of the personal meaning of their stories. The group generated discussions on the discrimination of mental health conditions in employment. Participants brainstormed ideas for the next topic ‘reconnecting’. This led to discussions around anger and coping strategies	5:0	36 (32-40)
9	*Exercise 6: Stories of strengths and weaknesses: ‘Reconnecting’* (As requested from the group, information was provided on local employment support groups.) Sharing and feedback on the topic. One member expressed being inspired by the storyteller. Members also shared their strategies in response to the storyteller’s struggle with anxiety. Discussions on reconnection beyond work and setbacks during recovery	5:0	36 (29-40)
10	*Ending and future* (As requested by the group, information about volunteering, employment and local mental health support groups were shared.) Flipcharts were put on display to assist reflection of the past ten weeks. Reflections include: a change in attitude towards mental health, feeling ‘normal’, knowing that they are not alone, being able to identify with other people and an increase in confidence, courage and perseverance. The facilitator read a written letter addressed to each participant’s contribution to group. Participants shared tips, inspirations and goals for the future	5:0	38 (34-40)

^a^The fifth participant joined at session three

^b^Session delivered by co-facilitator

#### Objective 5: Evaluation of Participant Response to Intervention

Changes in individual scores from baseline to follow-up were examined using within-subject analysis (Table 2). Outcome scores varied across participants. The only consistent trend was a drop in clinical symptoms from baseline to follow-up across all participants. Results were mixed for scores of recovery, insight, self-stigma and session rating scores. There was no clear trend between measures of recovery and insight. Only one participant’s score indicted a reliable improvement in self-stigma (bold figure in Table 2).

**Table 2:** Change scores (difference pre- and post-intervention)

Participant	Recovery^c^ (QPR total)	Self-stigma^d^ (BIS)	Insight total^c^ (ISMI-9)	Symptoms^d^ (BSI)
1	+ 5	− 0.07	− 1.52	− 15.92^b^
2	− 5	− 0.44	+ 1.14	− 7.08^b^
3	+ 9^b^	− 0.22	+ 2.28	− 6.6^b^
4	+ 22^b^	+ 0.06	+ 3.43^b^	− 45^b^
5^a^	+ 3	**− 0.78**^b^	+ 2.28	− 43^b^

^a^Baseline = Session 3

^b^Reliable change

^c^ + indicates improvement, − indicates deterioration

^d^ + indicates deterioration, − indicates improvement

Participants shared feedback which illustrates a positive response to the intervention and possibly supports the reduction of clinical symptoms from baseline to follow-up. One participant found ‘hearing people’s stories helped put difficult things into perspective’. Another participant shared changes in their engagement: ‘I didn’t come as I wasn’t sure whether it was helpful. I came back and picked it back up again’. Another participant reflected that ‘the group builds on your confidence in terms of sharing and gives you courage’. One participant shared ‘I got courage from the group…before there was something blocking me, but now everything is fine and I am very confident about it. Now I can join another group and wouldn’t be as worried…’.

Interestingly, participants as a group used new language to describe their mental health experiences, which were consistent with the above feedback. The language participants used seem to reflect a shift in identity and narrative e.g. ‘I am normal’, ‘I am not alone’, ‘Confidence’, ‘courage’, ‘perseverance’. It also corresponds with the recovery model’s suggestion of rebuilding a sense of self, belonging, identity, hope, and empowerment.

## Discussion

This study aimed to feasibility test the adapted narrative component of NECT for people with LOP. The findings are discussed with the five research questions.

### Research Question 1

What adaptations are required to apply the narrative module of NECT for people with LOP?

Pre-group consultations and facilitator-directed amendments during delivery and post-group feedback directed adaptations for the final manual (My Story v4). On reflection, these adaptations were a good fit for the characteristics of the targeted population. Firstly, cultural diversity is a common feature across cities in the UK and this was reflected in the different backgrounds of our group members. The changes to the readability of materials and mode of sharing addressed this diversity and allowed participants to fully express themselves. Secondly, the increased duration, telephone support, safety plans, and closed group recruitment enhanced the safety of the therapeutic space in the group. Symptoms such as persecutory delusions make it difficult for clients to feel safe and share personal information. Participants reflected that it was the first time they shared stories with people outside professional settings and their families.

Post-group feedback highlighted other areas of manual development. The recruited participants were mainly of working age which led to discussions around employment. It would be helpful to have flexibility in session topics, depending on the audience of the intervention. For example, retirement and social activity may be more relevant for older cohorts. Another area for development is the inclusion of individual therapists in risk management. Telephone support and safety plans were helpful but the communication between the researcher and the clinical team, i.e. individual therapist and care-coordinators was also essential in supporting participants.

### Research Question 2

Is the adapted module of NECT acceptable by people with LOP, their family/friends and clinicians?

Acceptability of the adapted intervention was reflected in no withdrawal, high attendance rate, and high engagement. Participants fed back that sessions increased their sense of hope, determination, willingness, openness, strength, courage, perseverance, control and choice. They also shared that it was a learning experience as they picked up other participants’ ways of coping. This is consistent with existing research findings of experiential learning and experiences of self (Roe et al. 2010). Participants’ keenness to continue and extend their recovery through support groups outside the service is a notable change compared to their initial fears and anxiety of joining groups. People in their social networks also observed these positive changes. It would be interesting to see whether this response to the intervention translates to the ‘very late-onset psychosis’ (VLOP) population, where onset is after 60 years of age (Howard et al. 2000).

### Research Question 3

Are outcome measures of NECT acceptable by people with LOP, their family members/friends and clinicians?

Completion of measures and qualitative feedback from participants indicated several issues with relevance, length, and wording, especially with QPR. The challenge is whether as a practitioner-researcher to (1) adhere to standardised measures so that data can be used for research or, (2) amend measures to reduce certain wordings that may trigger negative emotions in participants. These issues with measures were predicted in pre-group consultations and reflected by service users who developed QPR (Neil et al. 2009) but the decision was made to adhere to standardised measures as the key study objective was to assess feasibility including measures. To address concerns, facilitators were made aware of the issues and assisted the participants where possible. Participants were also given the option to opt-out. On reflection, adherence despite emotional impact raises fundamental questions around ethical issues of completing measures. Researchers may wish to examine whether the observed participant response to QPR in this study is replicated in wider-scale studies. If so, alternative measures or formal adaptations of QPR should be considered.

### Research Question 4

Is it feasible to deliver the adapted module of NECT within an outpatient NHS setting?

The successful delivery and positive responses suggest that this is feasible. However, the recruitment process was effortful. Individuals from both adult and older adult settings were approached. Poor referral and recruitment rates in older adult mental health services are not uncommon. Firstly, the cut-off age for a diagnosis of LOP remains debatable which makes it challenging for clinicians to consistently identify suitable clients. Secondly, the large geographical coverage of NHS trusts along with poor health and mobility in older adults doubles the difficulty clients experience in accessing this service. Finally, the combination of self-stigma, stigma from society and cultures (Burke et al. 2015) makes it difficult for help-seeking and for outreach services to connect with them.

### Research Question 5

Can the adapted module of NECT yield change in participants’ clinical, social and psychological recovery?

Although the sample size for this study is insufficient for making a valid suggestion, results and feedback appears to support the fact that the adapted intervention may promote recovery. Outcome scores varied across participants but there was one consistent trend in reduced clinical symptoms across all participants. Other measures especially scores on interpersonal and intrapersonal recovery were insignificant but show an emerging positive effect. It may be of interest to explore the drivers of this observed effect. The mechanisms of change for the original NECT lie in the interpersonal context, the process of sharing and narrating, and emotionally-present facilitators. (Yanos et al. 2012). It was thought that collective story-telling helps individuals reduce stigma, find new meaning, and create new narratives of themselves. The environment allows validation of experiences which is associated to hope, coping and recovery. Although the manual was adapted, these mechanisms of change were not altered.

Participants were initially reluctant to participate in discussions and share experiences but improved after the third session. On reflection, individuals were of different backgrounds and would unlikely have met within their pre-illness social circles or occupations. Despite this heterogeneity, participants were observed to slowly engage with each other. They became encouraging, validating and supportive in the final three sessions. It is interesting to note that each participant played a unique role in the group. For example, one client often shared insightful stories, another provided encouragement. This positivity and group cohesiveness is reflected in the zero withdrawal rate and high attendance. This result aligns with the recommendations to focus on group climate to improve attendance (Hansson et al. 2017).

Another mechanism of change is the involvement of emotionally-present facilitators. This is reflected in the emotional support provided outside of the group. Members have reflected during the group that they often felt unsupported even by close members of their family. Therefore, such emotional support may have helped sustain engagement and the successful delivery of the adapted intervention. Finally, self-stigma is a core component of NECT and the adapted intervention. Outcome scores of self-stigma showed reductions between baseline and follow-up for four of five participants. However, it is difficult to determine, given the small sample size, whether self-stigma is directly associated with recovery. Regardless, feedback illustrated changes in thoughts around self-stigma.

## Limitations

The generalisability of the current findings is limited to supporting feasibility rather than the effectiveness of the adapted intervention. The small sample size, restricted number of data points and lack of control/comparison group meant power may be reduced and there is a high risk of missing an effect. The use of proration in analysis inflates bias and internal consistency reliability (Schafer and Graham 2002). It would be crucial for future studies conducted on larger scales to adopt systematic methods to address missing data. Finally, the study adopted a modular approach to examine the narrative module specifically. From a research perspective, it is an advantage as one can gain an understanding of the effects of individual components of the intervention manual. From a clinical perspective, it would be difficult to generalize the findings as the stand-alone module cannot be used clinically as compared to the original standardised and complete manual.

## Implications

Research-wise, future studies need to compare the effectiveness of the original manual with the adapted manual. This will answer the fundamental question of whether an adaptation is required for the late-onset psychosis population. Clinical-wise, the findings from this sets the foundation for future studies to support the formation of a pathway and address this ‘forgotten’ population. The establishment of a formal pathway for late-onset psychosis population can reduce excess resources being spent on crisis services, GP visits, and physical health appointments, opening the opportunity for spending on other needs.

## Conclusion

This study systematically adapted and feasibility tested the narrative module of NECT for late-onset psychosis. Outcome results and qualitative feedback of the ‘My story’ intervention indicated it is feasible and acceptable for the targeted population. Further feasibility studies are warranted to confirm the suggested adaptations before a pilot trial is conducted to look at the effectiveness of the adapted intervention.
